# Meteorological Register

**Published:** 1845-06-01

**Authors:** 


*METEOROLOGICAL REGISTER.—Station, Buffalo Barracks, N. Y., North Latitude, 42 deg. 53 min.; West Longitude from Washington City, D.C. 1 deg. 53 min. 36 sec.—Altitude of Barometer above Lake Erie 96 feet.*

| 1845 | Barometer. | | | | Fahrenheit Thermometer. | | | | | Clearness of Sky | | | | Wind. | | | | Clouds. | | | | Wet Bulb | | Rain. | | |
|---|---|---|---|---|---|---|---|---|---|---|---|---|---|---|---|---|---|---|---|---|---|---|---|---|---|---|
| Ap'l | Sunrise. | 9 A. M. | 3 P. M. | 9 P. M. | Sunrise. | 9 A. M. | 3 P. M. | 9 P. M. | Daily Mean. | Sunrise. | 9 A. M | 3 P. M. | 9 P. M. | Sunrise. | 9 A. M. | 3 P. M. | 9 P. M. | Sunrise. | 9 A. M. | 3 P. M. | 9 P. M. | Sunrise. | 3 P. M. | Began. | Ended. | Q'ntity |
| 1 | 29 016 | 29 213 | 29 292 | 29 351 | 41 | 38 | 38 | 32 | 39 5 | 0 | 0 | 0 | 6 | s. w. 5 | s. w. 5 | s. w. 5 | s. w. 3 | n 0 | n e 2 | s w 3 | w 1 | 40 | 37 | 3 a. m. | 8 a. m. | 0 02 |
| 2 | 29 252 | 29 134 | 29 292 | 29 331 | 38 | 45 | 42 | 33 | 40 | 1 | 4 | 6 | 5 | s. 5 | s. w. 5 | s. w. 5 | s. w. 3 | s w 2 | s e 2 | w 3 | w 1 | 37 | 39 | 6 30 a. m. | 1 p. m. | 0 03 |
| 3 | 29 547 | 29 599 | 29 606 | 29 173 | 29 | 30 | 38 | 37 | 33 5 | 1 | 8 | 4 | 8 | n. w. 3 | w. 4 | s. w. 4 | s. w. 4 | n w 2 | w 1 | w 2 | n 1 | 27 | 37 | | | |
| 4 | 28 937 | 29 154 | 29 213 | 29 331 | 45 | 45 | 45 | 34 | 45 | 3 | 8 | 4 | 3 | n. w. 4 | w. 4 | s. w. 4 | s. w. 3 | w 3 | w 3 | n w 3 | w 2 | 47 | 46 | | | |
| 5 | 29 390 | 29 441 | 29 370 | 29 410 | 28 | 35 | 38 | 26 | 33 | 2 | 2 | 3 | 2 | n. w. 3 | n. w. 3 | s. w. 5 | s. w. 3 | n w 1 | w 2 | w 3 | w 1 | 26 | 37 | | | |
| 6 | 29 410 | 29 429 | 29 292 | 29 252 | 21 | 32 | 40 | 35 | 30 5 | 7 | 6 | 6 | 0 | e. 2 | s. 4 | s. w. 5 | s. w. 4 | n 0 | w 3 | w 3 | w 2 | 19 | 39 | | | |
| 7 | 29 284 | 29 292 | 29 331 | 29 419 | 26 | 28 | 36 | 24 | 31 | 2 | 0 | 0 | 0 | n. 2 | n. 4 | w. 3 | w. 3 | s w 1 | w 1 | w 1 | w 1 | 25 | 35 | 6 a. m. | 4 p. m. | 0 06 |
| 8 | 29 441 | 29 449 | 29 508 | 29 587 | 21 | 22 | 30 | 26 | 25 5 | 0 | 0 | 4 | 5 | n. w. 4 | w. 4 | n. w. 5 | w. 3 | n w 3 | w 2 | w 3 | w 2 | 19 | 29 | 5 a. m. | 11 a. m. | 0 04 |
| 9 | 29 587 | 29 606 | 29 508 | 29 232 | 20 | 32 | 36 | 35 | 28 | 4 | 7 | 0 | 6 | n. w. 2 | s. w. 3 | s. w. 4 | s. 6 | w 2 | w 1 | w 2 | w 1 | 19 | 36 | 7 30 p. m. | 8 p. m. | 0 12 |
| 10 | 28 906 | 28 906 | 28 977 | 29 139 | 41 | 44 | 45 | 39 | 43 | 0 | 2 | 1 | 0 | s. w. 3 | s. w. 3 | w. 2 | n. w. 4 | 0 0 | w 2 | w 1 | w 2 | 40 | 42 | | | |
| 11 | 29 232 | 29 390 | 29 469 | 29 488 | 35 | 36 | 43 | 32 | 39 | 1 | 2 | 9 | 10 | n. w. 3 | n. w. 5 | s. w. 4 | n. w. 3 | w 2 | w 3 | w 1 | 0 0 | 35 | 40 | | | |
| 12 | 29 587 | 29 634 | 29 626 | 29 555 | 27 | 36 | 44 | 39 | 35 5 | 6 | 7 | 3 | 1 | n. w. 2 | s. w. 4 | s. w. 4 | s. w. 3 | w 1 | w 1 | w 2 | w 1 | 28 | 40 | | | |
| 13 | 29 331 | 29 252 | 29 252 | 29 331 | 42 | 58 | 56 | 45 | 49 | 1 | 4 | 8 | 7 | s. w. 3 | s. w. 4 | s. w. 4 | s. w. 3 | w 2 | w 2 | w 1 | n 0 | 41 | 54 | | | |
| 14 | 29 449 | 29 528 | 29 488 | 29 469 | 37 | 47 | 50 | 38 | 43 5 | 5 | 8 | 9 | 9 | n. w. 3 | n. w. 3 | s. w. 3 | s. w. 3 | w 2 | 0 0 | 0 0 | 0 0 | 36 | 49 | | | |
| 15 | 29 257 | 29 528 | 29 488 | 29 370 | 36 | 46 | 58 | 59 | 47 | 4 | 9 | 6 | 8 | s. w. 3 | s. w. 3 | s. w. 3 | s. w. 3 | w 2 | n 1 | w 1 | s 1 | 35 | 55 | | | |
| 16 | 29 292 | 29 252 | 29 193 | 29 173 | 47 | 56 | 45 | 42 | 46 | 0 | 1 | 0 | 0 | n. e. 3 | n. e. 3 | n. e. 3 | n. e. 3 | n 2 | w 2 | w 1 | 0 0 | 46 | 45 | 1 p. m. | | |
| 17 | 29 213 | 29 252 | 29 331 | 29 351 | 39 | 43 | 57 | 46 | 48 | 0 | 0 | 0 | 0 | n. e. 3 | n. w. 3 | n. 3 | n. e. 3 | 0 0 | w 1 | 0 0 | s w 2 | 38 | 55 | | 8 a. m. | 0 65 |
| 18 | 29 410 | 29 421 | 29 410 | 29 394 | 44 | 53 | 70 | 51 | 57 | 0 | 0 | 4 | 1 | s. w. 2 | n. 3 | n. e. 2 | n. 2 | s w 2 | n 1 | w 2 | w 1 | 43 | 66 | | | |
| 19 | 29 331 | 29 331 | 29 272 | 29 331 | 48 | 57 | 52 | 44 | 50 | 0 | 0 | 0 | 0 | e. 2 | n. e. 2 | s. w. 2 | s. w. 2 | w 1 | w 2 | 0 0 | 0 0 | 47 | 51 | 1 a. m. | | |
| 20 | 29 331 | 29 370 | 29 390 | 29 410 | 42 | 46 | 44 | 42 | 43 | 1 | 0 | 0 | 0 | w. 2 | s. w. 3 | s. w. 3 | s. w. 3 | w 3 | w 2 | w 2 | w 1 | 41 | 44 | | 8 p. m. | 0 35 |
| 21 | 29 449 | 29 496 | 29 488 | 29 488 | 41 | 46 | 50 | 44 | 45 5 | 1 | 1 | 0 | 1 | n. w. 2 | n. w. 2 | n. w. 2 | e. 2 | n w 2 | n w 1 | n w 1 | e 1 | 49 | 49 | | | |
| 22 | 29 528 | 29 488 | 29 410 | 29 351 | 37 | 52 | 69 | 53 | 53 | 3 | 8 | 8 | 7 | n. e. 3 | s. e. 2 | n. 2 | e. 2 | e 1 | e 1 | n 1 | w 1 | 36 | 66 | | | |
| 23 | 29 351 | 29 331 | 29 331 | 29 299 | 57 | 67 | 82 | 68 | 69 5 | 4 | 6 | 6 | 6 | s. w. 4 | s. e. 3 | s. w. 1 | s. w. 3 | s e 2 | w 2 | w 1 | w 1 | 56 | 82 | 6 p. m. | 8 30 p. m. | 0 40 |
| 24 | 29 410 | 29 410 | 29 410 | 29 370 | 51 | 66 | 75 | 60 | 63 | 0 | 6 | 4 | 2 | s. e. 2 | s. w. 3 | s. w. 3 | s.w. 4 | 0 0 | w 2 | w 1 | w 2 | 59 | 72 | | | |
| 25 | 29 252 | 29 272 | 29 173 | 29 118 | 48 | 56 | 62 | 52 | 55 | 0 | 2 | 0 | 0 | n. e. 2 | n. w. 3 | n. e. 3 | s.w. 3 | w 3 | n 2 | w 3 | w 1 | 47 | 56 | 2 p. m. | 11 p. m | 0 75 |
| 26 | 29 213 | 29 213 | 29 213 | 29 134 | 45 | 51 | 72 | 56 | 58 5 | 3 | 8 | 5 | 6 | s. w. 3 | s. w. 3 | s. w. 3 | s.w. 3 | w 1 | w 1 | w 2 | 0 0 | 45 | 69 | | | |
| 27 | 29 252 | 29 252 | 29 311 | 29 311 | 59 | 60 | 67 | 48 | 63 | 4 | 1 | 2 | 0 | n. 1 | s. 3 | s. w. 3 | s.w. 3 | w 1 | w 3 | w 1 | w 1 | 51 | 66 | 7 a. m. | 8 a. m. | 0 04 |
| 28 | 29 449 | 29 469 | 29 508 | 29 488 | 44 | 60 | 55 | 57 | 49 5 | 4 | 1 | 1 | 0 | s. w. 2 | s. w. 2 | s. w. 3 | s·w. 3 | w 1 | w 1 | w 1 | 0 0 | 43 | 54 | | | |
| 29 | 29 547 | 29 559 | 29 567 | 29 567 | 42 | 55 | 66 | 52 | 54 | 5 | 6 | 7 | 7 | s. w. 1 | n. w. 3 | n. e. 4 | n. e. 3 | w 1 | w 2 | n w 2 | n 1 | 41 | 63 | | | |
| 30 | 29 547 | 29 523 | 29 441 | 29 449 | 50 | 62 | 77 | 66 | 63 5 | 3 | 0 | 6 | 0 | s. e. 3 | s. 4 | s. w. 3 | s.w. 3 | w 2 | w 1 | w 1 | 0 0 | 49 | 73 | 9 a. m. | 12 m. | 0 24 |
| M'n | 29 346 | 29 373 | 29 372 | 29 357 | 39 36 | 46 63 | 52 73 | 43 53 | 46 05 | 2 16 | 3 56 | 3 53 | 3 33 | | | | | | | | | 38 23 | 50 86 | | | 2 70 |

REMARKS.—1st—Rain, A. M.; 2d—Rain and snow, A. M.; 3d—High winds early A. M. No. 7, snow and rain at 6 P. M inappreciable; 4th—High winds all day and night; 5th—light snow, accompanied with high winds, snow inappreciable; 7th—Snow; 8th—snow; 9th—rain and hail; 10th—high winds at 7 30 P. M.; 16th—rain all night; 17th—rain, A. M.; 19th—began to rain at 1 A. M., showery all day; 20th—slight rain all day; 21st—trees budding; 23d—heavy rain, accompanied with thunder and lightning and high winds; 24th—trees in blossom; 25th—rain, accompanied with thunder and lightning, and very high winds; 26th—rainbow in east, extend from north to south at 6 P. M; 27th—showery; 28th—shower P. M. inappreciable; 30th—rain at 9 A. M. half an hour, heavy rain all night, accompanied with thunder and lightning.

EXPLANATIONS.—The amount of Clear Sky is designated by figures, as follows: 0 signifies no clearness of sky; 1 a very small portion, and so on to 10, which signifies entire absence of clouds or haze. The observations of Wind relate to direction and force; the former is expressed by the customary letters; the latter by figures from 0 to 10, as follows: 0 a calm; 1 barely appreciable breeze; 2 gentle breeze; 3 moderate do.; 4 brisk do.; 5 strong wind; 6 very strong do; 7 storm; 8 great do.; 9 hurricane; 10 violent do. The observations of clouds embrace direction and velocity; the latter is expressed relatively by numbers up to 10, as in the case of wind. The wet-bulb expresses the hygrometric condition of the atmosphere, as determined by evaporation of water upon the bulb of a thermometer covered with fine gauze. The numbers express the lowest point to which the mercury falls, by wetting the bulb and swinging the instrument in the open air. The figures under the head of rain express the vertical depth of water [whether rain or snow] in inches or fractions of inches.

